# 

**Published:** 1906-12-15

**Authors:** 


### ANNOUNCEMENTS.

SOUTHERN NEBRASKA DENTAL SOCIETY—The second meeting of the Southern Nebraska Dental Society was held in Superior, Neb. November 13th and 14th in Dr. Frank J. Nelson's office. A good attendance and a fine spirit. Several good clinics were given, also fine papers by Dr. J. M. Prime of Oxford, Dr. N. H. Morrison and Dr. E. A. Thomas of Red Cloud. The next meeting will be held in Superior in January. W. A. McHenry, Sec'y.

CHICAGO DENTAL COLLEGE ANNIVERSARY—Twenty-fifth Anniversary Reunion, Celebration and Clinic of the Chicago College of Dental Surgery Alumni Association.

On the sixteenth and seventeenth days of January; 1907, the Alumni Association of the Chicago College of Dental Surgery will celebrate the twenty-fifth anniversary of the establishment of the College by holding a grand reunion and clinic. Arrangements have been made for a number of papers, a very extensive clinic, a theater party and a banquet. A railroad rate of a fare and a third for the round trip from all points in the United States and Canada on the certificate plan has been arranged for.

A cordial invitation is extended to the general profession to be present, as well as all members of the Alumni Association and all graduates of the College.

R. C. Brophy, J. P. Buckley, } *Com. on Publicity.*

---

American Society of Orthodontia—The next meeting of this Society will be held at the New Astor Hotel in New York City, Dec. 27th to 29th., 1906—Dr. F. S. McKay, Sec'y.

---

Institute of Dental Pedagogics—Preparations for the Fourteenth Annual Meeting of the Institute are practically complete and the outlook is most favorable for an exceptionally instructive session. Those who have attended the Institute in the past appreciate its importance and the value of its proceedings to Dental Education—equaled by no other body. Those who have never attended cannot be too strongly urged to be present at the coming meeting, being certain to be more than repaid for any sacrifice made in the effort.

The Executive Committee have secured the following essayists, whose papers will be discussed by eminent men from all parts of the country:

1 President's Address..............*Dr D. R. Stubblefield, Nashville.*
2 The Teachings of Anesthesia, The Prevention and Treatment of Emergencies, (Second paper in series: First in New

York, 1905)..................... *Dr. Herman Prinz, St. Louis.*
3 Teaching of Operative Technics *Dr. D.M.Cattell Nashville.*
4 Prosthodontia as taught in Tufts
College Dental School............ *Dr. Jos. King Knight, Boston.*
5 A Method of Teaching Porcelain
Technic.................................. *Dr. J.Q. Byram, Indianapolis.*
6 A Method of Teaching
Orthodontia ................ *Dr. H. T. Smith, Cincinnati.*
7 The Teaching of Materia Medica
and Therapeutics......................... *Dr. J. P. Buckley, Chicago*
8 Report of Committee on Dental
Nomenclature................................ *Dr. S. H. Guilford, Phila.*

The exhibits will be in charge of Dr. H. E. Friesell, who will give personal attendion to gathering as large and instructive exhibits from each College as possible and we bespeak in his behalf the full co-operation of every College in seeing that such exhibit is forwarded in due time.

The local Committee, comprising Drs. Goslee, Gilmer and Gallie have arranged for the welfare of the Institute at the Palmer House, where every courtesy possible will be shown those attending.

The rates will be $1.50 for one person and $2.00 for two without a bath, $2.50 for one and $.4.00 for two with a bath.
D. R. Stubblefield, *President*, W. E. Willmott, *Secretary*
Ellison Hillyer, *Chairman Executive Board.*

—✦—

Fred E. Dodge, D.D.S. formerly of Vienna, announces his location at Universitaets Platz 5, Strassburg Alsace, Successor of Dr. I. R. Westervelt.

